# Data on roof renovation and photovoltaic energy production including energy storage in existing residential buildings

**DOI:** 10.1016/j.dib.2022.107874

**Published:** 2022-01-26

**Authors:** Delia D'Agostino, Danny Parker, Paco Melià, Giovanni Dotelli

**Affiliations:** a European Commission, Joint Research Centre (JRC), Ispra (VA), Italy; b Florida Solar Energy Center, University of Central Florida, USA; c Politecnico di Milano, Dipartimento di Elettronica, Informazione e Bioingegneria, Milan, Italy; d Politecnico di Milano, Department of Chemistry, Materials and Chemical Engineering, Milan, Italy

**Keywords:** Roof insulation, Energy efficiency, PV systems, Residential building retrofit, Building energy simulations

## Abstract

This data article refers to the paper "Optimizing photovoltaic electric generation and roof insulation in existing residential buildings” [1].

The reported data deal with roof retrofit in different types of existing residential buildings (single-family, multi-family and apartment complex) located in Milan (Northern Italy). The study focus on the optimization of envelope insulation and photovoltaic (PV) energy production associated with different building geometries, initial insulation level, roof constructions, and materials.

The data linked within this article relate to the modelled building energy consumption, renewable production, potential energy savings, and costs. Data refer to two main scenarios: refurbishment (roof in need of replacement and insulation) and re-roofing (energy intervention for roof improvement). Data allow to visualize energy consumption before and after the optimization, selected insulation level and material, costs and PV renewable production (with and without energy storage). The reduction of energy consumption can be visualized for each building type and scenario. Further data is available on CO_2_ emissions, envelope, materials, and systems.

## Specification Table

**Table:** 

Subject	Energy.
Specific subject area	Energy retrofit of the roof.
Type of data	Building simulation file.
How data were acquired	Data were processed using the BEopt tool.
Data format	.BEopt
Parameters for data collection	Data of performance calculations and dynamic simulation modelling of existing residential buildings.
Description of the data collection	Data collected from different sources for the model set up (e.g. weather data files, Eurostat cost data, market surveys, literature, available information on technological measures, Standards), then processed by BEopt.
Data source location	Table 1, Table 2, Table 3 of [1] summarize the primary data sources used.
Data accessibility	Data are provided in supplementary materials directly with this article.
Related research article	Delia D'Agostino, Danny Parker, Paco Melià, Giovanni Dotelli, “Optimizing photovoltaic electric generation and roof insulation in existing residential buildings”, Energy and Buildings, 255 (2022) 111652, https://doi.org/10.1016/j.enbuild.2021.111652.

## Value of the Data

• The data provide quantitative information on roof retrofit in different existing building prototypes;
• The data show modelled energy consumption, optimal insulation level, renewable production, primary energy savings, and costs;
• Energy and economic data related to different retrofit options and PV production guide how to optimize roof retrofit;
• The data can be useful for the development of specific measures and incentives related to roof, comparison with other building types, other retrofit intervention, or further analysis.
• The data support the Green Deal and the Renovation Wave initiative to boost renovation at European level [2].

## 1 Data Description

The data provided in this paper are the developed file that documents the modeling process supporting the research. To optimize roof insulation and determining the cost-effectiveness of installing PV (with and without energy storage) in different building prototypes, a simulation-based optimization model has been developed. The methodology and the research assumptions are reported in Section 2 of [1]. A total of 120 simulations (40 per building type) were carried out for three baseline building prototypes (single-family, multi-family, apartment complex). Both pre- and post-intervention consumption is based on simulation. Pre-intervention consumption was compared to typical consumption as evaluated by the Danish Building Institute and Ecofys described in Section 4.1 of paper [1]. Once we had good agreement, we simulated buildings as if they were all electric as this will be the direction of the housing energy supply in the future. The modelled building prototypes and its main properties are detailed in Section 2.2 of [1], while roof characteristics are in Section 2.3 of [1]. The economic parameters and assumptions are in Section 2.4 of [1].

The shared data are the building simulations files carried out using the software BEopt. These data detect the optimal building retrofit design considering different level of insulation, materials, costs, PV energy production, with and without energy storage. Data refer to existing residential buildings and include the main building characteristics (efficiency measures, envelope, systems, technologies, lighting, renewables).

As detailed in [3], BEopt is a widely-recognized optimization software that implements a sequential search technique to optimize the building design starting from a base configuration. It has the EnergyPlus and TRNSYS engines to perform the dynamic simulations of the building. In more details, EnergyPlus calculates hourly household needs (heating, cooling, water heating and appliance), while TRNSYS estimates the renewable energy production.

Hourly Typical Meteorological Year (TMY) data files has been included in the model for the city of Milan for the period 2004-2018. Table 1 shows average monthly mean temperature, relative humidity, and precipitation and sunshine hours along the year for Milan.

**Table 1:** Climate in Milan: monthly mean temperature, relative humidity, sunshine hours, precipitation.

Month	Jan	Feb	Mar	Apr	May	Jun	Jul	Aug	Sep	Oct	Nov	Dec
Temperature (°C)	5.9	9.0	14.3	17.4	22.3	26.2	29.2	28.5	24.4	17.8	10.7	6.4
Relative humidity (%)	86.0	78.0	71.0	75.0	72.0	71.0	71.0	72.0	74.0	81.0	85.0	86.0
Sunshine (hours)	58.9	96.1	151.9	177.0	210.8	243.0	285.2	251.1	186.0	130.2	66.0	58.9
Precipitation (mm)	58.7	49.2	65.0	75.5	95.5	66.7	66.8	88.8	93.1	122.4	76.7	61.7

For the baseline building, provided data include selected energy efficient measures, related to envelope, appliances and systems. This comprises technical features as well as life expectancy, operation, maintenance, and replacement costs [4]. Incremental and cumulative costs can be visualized as well. The shared data include both the model input and output.

Provided data allow the identification of the cost-optimal insulation level within the cost-optimal curve that reports global costs (€/m^2^) and energy consumption (kWh/m^2^y).

**Fig. 1:**
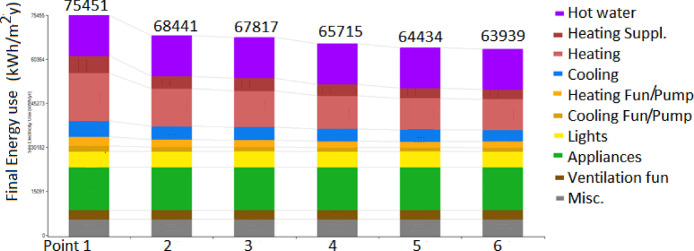
Data for the Milan case from BEopt/EnergyPlus simulation showing predicted energy use for each evaluated insulation increment in the multi-family building. Colors represent different energy end-uses. Heating (red), cooling (blue) and associated fans are the end uses that are strongly impacted. Point 1 is no insulation. Point 2 is a low insulation (0.80 (W/m^2^K), Point 3 is a medium level (0.60 W/m^2^K) and 4, 5 and 6 are high (0.35 W/m^2^K), very high (0.20 W/m^2^K) and extra high levels (0.15 W/m^2^K). Raw data from this figure are attached to the paper.

Data outputs can be visualized from the provided material in different forms: energy consumption, savings, costs and renewable production. An example of energy data visualization across insulation levels is shown in Fig. 1. The data reported in the figure are made available as hourly data in the provided Excel spreadsheet where the following columns are reported: Base (kWh), Insulated (kWh) and PV (kWh). Data relate the final energy use in the building at each iteration for the different energy uses (e.g. heating, cooling, hot water). In the output section, all consumption are available from the starting building configuration.

## 2 Experimental Design, Materials and Methods

Table 2 summarizes the data of the optimization modelling carried out for each building and roof type, and PV (with and without energy storage) (Table 1 – Table 4 of [1]). They are provided for each scenario (refurbishment and re-roofing), building type and initial insulation level (no or low insulation). For each building type, the table indicates the optimal insulation level identified by the algorithm, the amount of energy consumed pre- and post- intervention, the energy output of the PV system, the reduction in CO_2_, the incremental costs, and the annual costs pre- and post- intervention. These values are normalized per building floor area and per occupant.

**Table 2:** Optimized insulation levels and PV outputs by building type.

Building prototype	Parameter	No Existing Insulation		Low Existing Insulation	
		Refurbish	Re-roof	Refurbish	Re-roof
Single-family	Optimal Insulation (W/m^2^K)	0.20 (Very high level)	0.20 (Very high level)	0.20 (Very high level)	0.20 (Very high level)
	Pre- intervention (kWh)	11867	11867	10589	10589
	Post-intervention (kWh)	9853	9853	9853	9853
	Rooftop PV (kWh)	7488	7488	7488	7488
	Pre-intervention (kWh/m^2^)	98.9	98.9	88.2	88.2
	Post-intervention (kWh/m^2^)	19.7	19.7	19.7	19.7
	Primary Energy savings (%)	80.1	80.1	77.7	77.7
	CO_2_ Reduction (t/year)	4.6	4.6	4.0	4.0
	Incremental cost (€)	13010	15908	12740	15638
	Annual cost pre – intervention (€)	3066	3066	2812	2812
	Annual cost post-intervention (€)	1516	1629	1527	1640
Multi-family	Optimal Insulation (W/m^2^K)	0.20 (Very high level)	0.20 (Very high level)	0.20 (Very high level)	0.20 (Very high level)
	Pre- intervention (kWh)	75451	75451	68441	68441
	Post-intervention (kWh)	64379	64379	64377	64377
	Rooftop PV (kWh)	37462	37462	37462	37462
	Pre-intervention (kWh/m^2^)	78.9	78.9	71.6	71.6
	Post-intervention (kWh/m^2^)	28.2	28.2	28.2	28.2
	Primary Energy savings (%)	64.3	64.3	60.7	60.7
	Incremental Cost (€)	59078	74780	57613	72955
	CO_2_ Reduction (t/year)	23.7	23.7	20.3	20.3
	Annual Cost Pre – intervention (€)	20330	20330	18962	18962
	Annual Cost Post-intervention (€)	11022	13328	11003	11742
Apartment Complex	Optimal Insulation (W/m^2^K)	0.35 (High level)	0.35 (High level)	0.35 (High level)	0.35 (High level)
	Pre- intervention (kWh)	295991	295991	278936	278936
	Post-intervention (kWh)	272817	272817	272817	272817
	Rooftop PV (kWh)	146674	146674	146674	146674
	Post-intervention (kWh/m^2^)	73.0	73.0	68.8	68.8
	Post-intervention (kWh/m^2^)	31.1	31.1	31.1	31.1
	Primary Energy savings (%)	57.4	57.4	54.8	54.8
	CO_2_ Reduction (t/year)	83.1	83.1	74.7	74.7
	Incremental Cost (€)	268842	309214	257774	298146
	Annual Cost Pre – intervention (€)	76554	76554	72342	72342
	Annual Cost Post-intervention (€)	45376	46945	45018	46857

Table 3 reports the results of the building prototypes and scenarios that include PV systems with electrical storage. The Table indicates energy savings, costs and optimal insulation in the two scenarios, as in Table 1 but including the electrical storage. The installed electrical storage was 4 kWstorage/kW PV. This is detailed in Section 4.3 and Table 4 of paper [1]. Storage is 24 kWh for a 6 kW PV system on residential building and scales with the installed PV on the other systems [5], [6], [6]. This is larger than might be considered for non-electric buildings [8], [9], [10], but this level was deemed necessary for effective daily storage for the transformation of heating and water heating from natural gas to electric systems [5], [6], [7], [8], [9], [10], [11], [12], [13].

**Table 3:** Insulation optimization and PV with energy storage.

Building prototype	Parameter	No Existing Insulation		Low Existing Insulation	
		Refurbish	Re-roof	Refurbish	Re-roof
Single Family	Optimal Insulation (W/m^2^K)	0.15 (Extra high level)	0.15 (Extra high level)	0.15 (Extra high level)	0.15 (Extra high level)
	Pre- intervention (kWh)	11867	11867	10589	10589
	Post-intervention (kWh)	9780	9780	9780	9780
	Rooftop PV (kWh)	7222	7222	7222	7222
	Pre-intervention (kWh/m^2^)	98.9	98.9	88.2	88.2
	Post-intervention (kWh/m^2^)	21.3	21.3	21.3	21.3
	Primary Energy savings (%)	78.4	78.4	75.8	75.8
	CO_2_ Reduction (t/year)	4.6	4.6	3.9	3.9
	Incremental Cost (€)	33637	36536	33367	36265
	Annual Cost Pre – intervention (€)	3066	3066	2812	2812
	Annual Cost Post-intervention (€)	3629	3742	3640	3753
Multi-family	Optimal Insulation (W/m^2^K)	0.20 (Very high level)	0.20 (Very high level)	0.20 (Very high level)	0.20 (Very high level)
	Pre- intervention (kWh)	75451	75451	68441	68441
	Post-intervention (kWh)	64379	64379	64379	64379
	Rooftop PV (kWh)	36571	36571	36571	36571
	Pre-intervention (kWh/m^2^)	78.9	78.9	71.6	71.6
	Post-intervention (kWh/m^2^)	29.1	29.1	29.1	29.1
	Primary Energy savings (%)	63.1%	63.1%	59.4%	59.4%
	Incremental Cost (€)	149110	165062	147645	163347
	CO_2_ Reduction (t/year)	23.3	23.3	19.9	19.9
	Annual Cost Pre – intervention (€)	20330	20330	18962	18962
	Annual Cost Post-intervention (€)	18704	19315	18716	19326
Apartment Complex	Optimal Insulation (W/m^2^K)	0.20 (Very high level)	0.20 (Very high level)	0.20 (Very high level)	0.20 (Very high level)
	Pre- intervention (kWh)	295991	295991	278936	278936
	Post-intervention (kWh)	270293	270293	270293	270293
	Rooftop PV (kWh)	143186	143186	143186	143186
	Pre-intervention (kWh/m^2^)	73.0	73.0	68.8	68.8
	Post-intervention (kWh/m^2^)	31.3	31.3	31.3	31.3
	Primary Energy savings (%)	57.1	57.1	54.4	54.4
	CO_2_ Reduction (t/year)	82.6	82.6	74.2	74.2
	Incremental Cost (€)	705225	745597	694156	734528
	Annual Cost Pre – intervention (€)	76554	76554	72342	72342
	Annual Cost Post-intervention (€)	80683	82252	80325	81893

**Fig. 2:**
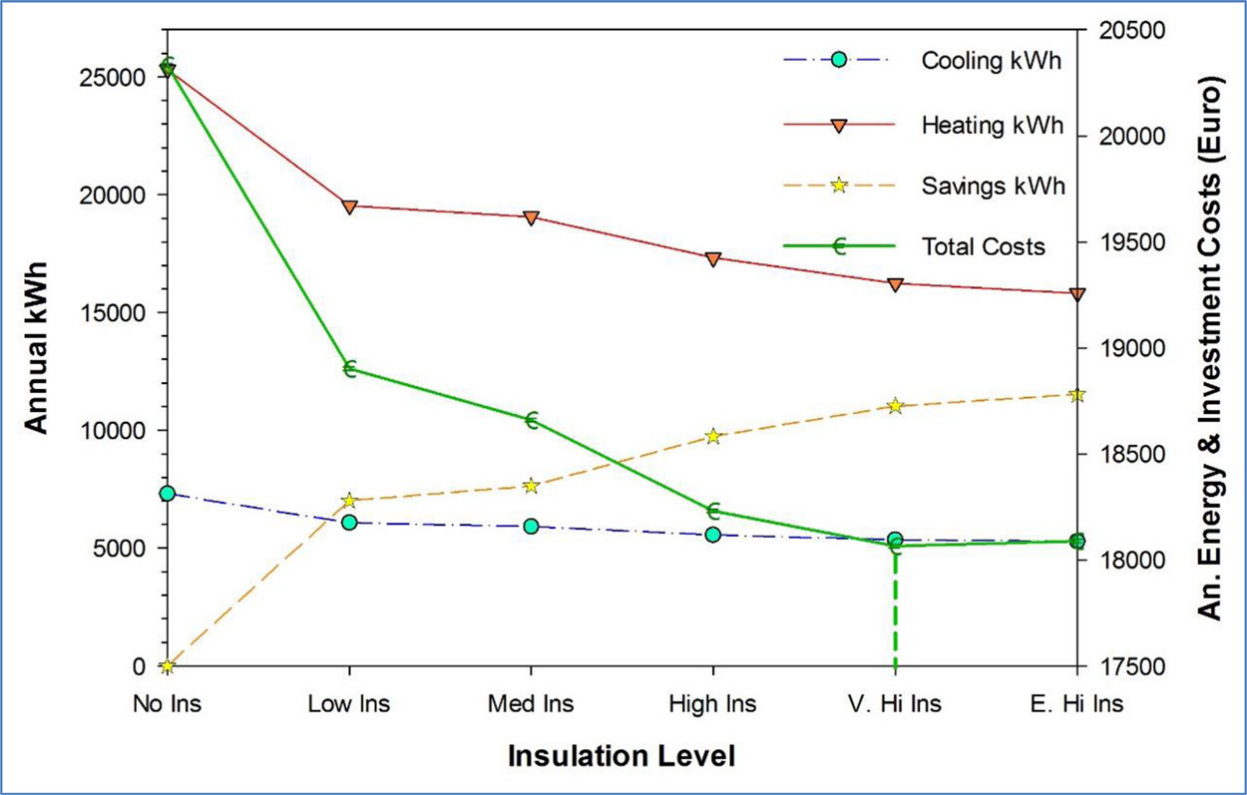
Illustration of insulation optimization for multi-family building in Milan (figure based on data in Table 1).

Fig. 2 shows an illustration of the data that can be visualized for the insulation optimization process related to the multi-family building. On the x-axis are the analyzed insulation levels from no insulation to extremely high insulation. The y-axis shows simulated kWh per year for heating (red) and cooling (blue) as well as the produced savings at each level (yellow). On the right axis are the total annual costs of the refurbishment investment and energy costs. The very high insulation level (0.20 W/m^2^K) is identified as the optimal least cost increment as detailed in [1].

## Ethics Statements

No ethics fields involved.

## CRedit Author Statement

Work plan: **Delia D'Agostino:** Simulations and result analysis, Writing, Revision; **Danny Parker:** Simulations and result analysis, Writing, Revision; **Paco Melià:** Revision, **Giovanni Dotelli:** Revision.

## Declaration of Competing Interest

The authors declare that they have no known competing financial interests or personal relationships that could have appeared to influence the work reported in this paper.

## Appendix Supplementary materials

Image, application 1

Image, application 2
